# Acute manic state with psychotic features in a teenager with autoimmune encephalitis: a case report

**DOI:** 10.1186/s13256-021-02879-2

**Published:** 2021-05-31

**Authors:** Sara Wallengren, Björn Axel Johansson, Olof Rask

**Affiliations:** 1 Region Skåne, Child and Adolescent Psychiatry, Regional Inpatient Care, Emergency Unit, Malmö, Sweden; 2 Department of Clinical Sciences Lund, Division of Child & Adolescent Psychiatry, Lund University, Lund, Sweden; 3 Department of Clinical Sciences, Division of Pediatrics, Lund University, Lund, Sweden

**Keywords:** Adolescent, Anti-*N*-methyl-d-aspartate receptor encephalitis, Bipolar disorder, Clinical decision-making, Psychotic disorders

## Abstract

**Introduction:**

Autoimmune disorders have become increasingly acknowledged as having a more causative role in encephalitis than previously assumed. Anti-*N*-methyl-d-aspartate receptor encephalitis seems to be the most prevalent disorder. Symptoms of the neuropsychiatric phase in children and adolescents include abnormal behavior, seizures, and neurologic symptoms. We present a report on a teenage girl with predominantly psychiatric symptoms, highlighting the need for awareness of the disease and multidisciplinary collaboration.

**Case presentation:**

Our patient, a 17-year-old girl of Middle Eastern origin, had no earlier medical history, but a family history of autoimmune disease. One morning, she could not recognize her mother and soon developed increased energy and pressured speech. The condition worsened, with paranoid delusions. In the emergency unit, she ran around speaking incoherently. The condition was interpreted as a full-scale mania. After pediatric clearance, the patient was admitted to the Department of Child and Adolescent Psychiatry. Mood-stabilizing treatment was initiated with second-generation psychotics and lithium, but this brought no improvement. A multidisciplinary discussion was held with physicians from psychiatry and neurology. A lumbar puncture showed *N*-methyl-d-aspartate receptor antibodies, and autoimmune treatment was initiated. Computed tomography thorax/abdomen revealed a right-sided ovarian tumor. After salpingo-oophorectomy, our patient’s mental status gradually improved, as demonstrated by repeated testing. Seven months post discharge she was in a stable relationship and performing well in school.

**Conclusion:**

This case underlines the importance of collaboration between child and adolescent psychiatry and pediatrics, and gives pointers for timely diagnosis. Manic adolescents who do not respond to mood-stabilizing treatment should be subjected to further consultations and investigations. Psychiatrists and neurologists should develop an integrated approach to the management of brain disorders.

## Background

It has been proposed that autoimmunity could play a causative role in a number of neuropsychiatric disorders, including psychosis [1]. Autoimmune conditions have also become increasingly recognized as causes of encephalitis [2]. Diagnostic tools, such as assays for neurotrophic autoantibodies, are becoming readily available for clinicians, that is, psychiatrists and neurologists. This shifting paradigm in clinical neuropsychiatry is illustrated by this case.

Anti-*N*-methyl-d-aspartate receptor (anti-NMDAR) encephalitis was first described a decade ago [3]. Our understanding of this disease entity has since increased, thanks to basic neuroscientific research and accumulated clinical data from published reports. Several other antineuronal autoantibodies associated with autoimmune encephalitis have subsequently been detected, but anti-NMDAR encephalitis seems to be the most prevalent disorder. Recent reports now indicate that anti-NMDAR encephalitis may surpass the incidence of viral etiologies in young adults [4].

In anti-NMDAR encephalitis, pathogenic immunoglobulin G (IgG) antibodies bind to the GluN1 subunit of NMDARs, causing receptor internalization and neuronal network dysfunction [5]. First reported as a paraneoplastic phenomenon, it has now been established that both malignancy and infections may trigger antibody formation. The frequency of an underlying tumor (in most cases an ovarian teratoma) varies with age and sex, ranging from 0% to 4% in children younger than 12 years, to 58% in women over 18 years [6].

Anti-NMDAR encephalitis is a multistage illness that worsens over time if untreated. A typical illness course often involves three or four stages: a prodromal phase resembling a viral infection, an early neuropsychiatric phase characterized by neurological symptoms, such as seizures and/or behavioral changes, followed by a late phase with further clinical deterioration, including unresponsiveness, hyperkinesia, and dysautonomia. Intensive care support may be required, and reported mortality rates with aggressive treatment are 4% [7].

Recently published consensus criteria state that an anti-NMDAR encephalitis diagnosis can be made after reasonable exclusion of other disorders, when IgG anti-GluN1 antibodies are detected in CSF and in the presence of rapid onset of symptoms from one or more of the following groups: abnormal (psychiatric) behavior or cognitive dysfunction, speech dysfunction, seizures, movement disorder, decreased level of consciousness, and autonomic dysfunction. Laboratory findings such as abnormal EEG and/or cerebrospinal fluid (CSF) with pleocytosis or oligoclonal bands are also mentioned in the guidelines to aid diagnosis [8].

A defining pathognomonic feature is still lacking, and the great variability in symptoms makes the diagnosis of anti-NMDAR encephalitis challenging. Symptom presentation has been reported to be “more neurological in children,” and “more psychiatric in adults,” showing this disease to be at the interface of the fields of neurology and psychiatry [6]. We report on a case of a teenage girl with predominantly psychiatric symptoms that are described in detail, which highlights the need for awareness of the disease entity and for multidisciplinary collaboration.

## Case presentation

Our patient, a 17-year-old girl born in Sweden but with Middle Eastern origin, had no earlier medical history. She was brought up in a home where physical violence, before the parents separated, was frequently present. Our patient’s grandmother suffered from depression, diabetes, and rheumatoid arthritis. In her late fifties, her personality altered, with confusion and elevated mood. A lumbar puncture was performed that, according to our patient’s mother, revealed pathological signs. Shortly afterwards, she developed epileptic seizures and died at the age of 57. No autopsy was performed. Our patient’s mother had suffered from unipolar depressions. Our patient’s first contact with the child and adolescent psychiatry services was 5 years before admission for disruptive behavior. A broad psychiatric evaluation was scheduled, but failed as the family did not show up to the planned appointments. At the time of admission, our patient had a boyfriend and was performing well on a business program at high school, receiving assistance due to dyslexia. She lived with her mother and her two younger siblings. Prior to admission, both our patient and her boyfriend had experienced symptoms from viral infections.

On the morning 3 days before admission our patient, to her mother’s surprise but soon despair, asked: “Who are you?” Our patient soon developed increased energy and pressured speech. The condition worsened, with insomnia and paranoid delusions, and her mother called 911. In the pediatric emergency room (ER), the patient ran around shouting, talking incoherently, removing her clothes, and approaching the staff in a provocative manner. Blood tests and a CT head scan showed no significant abnormalities. Drug screening, including testing for ethanol, was negative. The neurological examination showed no irregularities. The patient’s condition was interpreted as a full-scale mania. A senior consultant from the Department of Child and Adolescent Psychiatry made the same assessment later the same day. A decision was made on coercive care. After pediatric clearance, the patient was admitted to the Department of Child and Adolescent Psychiatry.

The paranoid delusions persisted at the emergency unit of the Department of Child and Adolescent Psychiatry. Mood-stabilizing treatment was initiated, with second-generation antipsychotics (olanzapine) and lithium, and benzodiazepines (lorazepam, oxazepam) were administered on several occasions. The doses were up-titrated in the following days. The patient’s speech remained incoherent, and she was confused and presented paranoid delusions. EEG and MRI of the brain showed normal findings, but a second review of the electroencephalography (EEG) later revealed unspecific regional slowing. Lithium concentrations were taken regularly, and despite therapeutic levels, no improvement was seen. Our patient continued to be labile, with memory loss, hyperactivity, and delusions. Signs of possible autonomic dysregulation, fever, and tachycardia were observed, along with tremor. Neuroleptic malignant syndrome (NMS) was suspected, and the patient’s vital parameters were monitored along with an olanzapine taper. The patient’s neurocognition was monitored using mini-mental state examination (MMSE)/Montreal Cognitive Assessment (MOCA).

A multidisciplinary discussion concerning etiology and treatment was held with physicians from psychiatry and neurology. Electroconvulsive therapy (ECT) was considered as a possible treatment intervention, and whether the psychiatric picture could represent an episode of acute encephalitis was discussed. To test the hypothesis, a lumbar puncture under full anesthesia was performed. The results showed moderately increased cerebrospinal fluid (CSF) pressure (34 cm water/25 mm Hg), minor pleocytosis and increased level of albumin. Two days later, NMDAR-antibodies of IgG-isotype were detected with strong positivity (titer 1/100) in cerebrospinal fluid using immunofluorescence microscopy on transfected cells (commercial assay; Euroimmun, Lübeck, Germany). Our patient was transferred to the Department of Pediatrics for continuous collaborative care, with frequent psychiatric evaluations.

The patient remained psychotic and was still under coercive care. Autoimmune treatment with intravenous immunoglobulin (IVIG; 0.4 g/kg/day for 5 days) combined with methylprednisolone (1 g/day for 5 days) was initiated. A computed tomography (CT) of the thorax/abdomen revealed a right-sided ovarian tumor 13 cm in diameter, with no signs of spreading. In preparation for surgery, lithium and olanzapine were tapered off. Tumor markers before surgery showed beta human chorionic gonadotropin (β-hCG) <1 and alpha-fetoprotein (AFP) 209 units (reference < 10). Our patient underwent a right-sided salpingo-oophorectomy. The biopsy showed an immature teratoma (stage IA, grade 3). After surgery, AFP was normalized. The patient’s mental status slowly improved, as evaluated with a mini-mental state examination (MMSE) and a Montreal Cognitive Assessment (MOCA) test (Table). Initial MMSE testing had resulted in 18/30 points, indicating severe cognitive impairment, with difficulties in orientation, attention/calculation, memory, and complex commands (Table 1).

**Table 1:** Cognitive assessment after start of autoimmune therapy

Category	PP, MMSE^a^	Day 0	Category	PP, MOCA	Day 2	Day 5	Day 7	Day 19	7 months
Orientation to time	5	3	Visuospatial/executive ability	5	5	3	3	3	5
Orientation to place	5	4	Naming	3	3	2	3	2	3
Registration	3	3	(Memory)^b^	0					
Attention and calculation	5	1	Attention	6	3	6	6	5	5
Recall	3	1	Language	3	2	2	2	2	2
Language	2	2	Abstraction	2	1	0	2	1	2
Repetition	1	1	Delayed recall	5	3	4	5	4	5
Complex commands	6	3	Orientation	6	6	6	6	6	6
Total points	30	18	Total points	30	23	23	27	26	28

Cognitive assessment, number of days after start of autoimmune therapy.* PP*, possible points; *MMSE*, mini-mental state test; *MOCA*, Montreal Cognitive Assessment

^a^MMSE only performed at the first evaluation, thereafter MOCA

^b^Memory gives no points and is included in “delayed recall”

It was decided that our patient should be treated for the malignancy aligned with the children’s oncology protocol postulating no up-front cytostatic treatment after surgery. Sequential follow-up with testing for tumor markers (β-hCG and AFP) and vaginal ultrasound was recommended. Our patient was granted temporary leave from the hospital 5 weeks after admission. Oral steroid treatment (prednisolone) was tapered off over 4 weeks. At the 3-month post-discharge check-up, our patient reported irregular menstruations, increased appetite, disturbing striae, and numbness of fingers.

During a return visit to the emergency unit of the Department of Child and Adolescent Psychiatry 7 months post-discharge, our patient was stable. She recognized the staff, and was friendly and accommodating. She related well to her mother. Her basic mood was neutral, and no signs of psychosis nor suicidal ideations were seen. On MOCA she now scored 28/30 points, with deductions for subtraction and word flow. Our patient reported a complete amnesia from her stay at the hospital and 3 days prior to admission. She lived with her mother and had caught up with schoolwork, receiving a grade A in Swedish. Our patient reported that she experienced sequelae in form of visual impairment in her right eye and lingering obesity related to previous cortisone treatment, but had reduced weight from 100 kg to 90 kg. She was in a stable relationship with her boyfriend, who she had met before she became sick. Our patient told us that, earlier in the year, she had accidentally become pregnant and had an abortion. She had plans for her future that included getting a drivers’ license, and after graduation from school she began working in the boyfriend’s hometown. A medical follow-up program was set up with the gynecologic oncology service.

## Discussion and conclusions

We present a clinical report on a teenage patient with symptoms resembling an acute manic state with psychotic features, subsequently diagnosed with anti-NMDAR encephalitis. It is the authors’ experience that the diagnosis is still rare in clinical practice, and that emergency mental health care departments should make the necessary adaptions to allow optimal assessment and management. This case illustrates the need for increased awareness of possible autoimmune etiology in neuropsychiatry, gives pointers for timely diagnosis, and suggests methods of monitoring neurocognitive symptoms.

Early diagnosis and treatment are important, to reduce symptom escalation and prevent hippocampal damage [9]. Our patient presented with strict psychiatric symptoms early in the course of the disease, and was brought to the psychiatric emergency unit after initial pediatric clearance. The family history of psychiatric disease increased the challenge of correct diagnosis. However, our patient also had a family history of autoimmune disease, a finding we have not previously seen reported in anti-NMDAR encephalitis, but could indicate a predisposition for autoimmune disease. In a retrospective review of patients with autoimmune encephalitis, those found to be positive for NMDAR-antibodies (*n* = 53, mean age 30 years) often first had viral encephalitis as a working diagnosis, suggested by the clinical picture, acute neurological changes, and CSF pleocytosis [10]. The authors suggested some clinical signs that could serve as “warning flags” possibly assisting in earlier diagnosis, including seizures, catatonia, autonomic instability, or hyperkinesia. Specific signs in adolescent patients with this condition were, however, not described.

Our patient showed sensitivity to neuroleptics, and displayed mild dysautonomia symptoms. In anti-NMDAR encephalitis, sensitivity to neuroleptics and/or symptoms mimicking neuroleptic malignant syndrome has been described, and this should thus arouse suspicion [11]. She did not respond to symptomatic treatment as expected, and showed a broad pallet of psychiatric symptoms. A prodromal history of flu-like symptoms was also reported. These circumstances led to investigations for (autoimmune) encephalitis and testing for pathogenic antibodies. Neuroimaging and EEG were initially interpreted as normal in our patient, but when reviewed the EEG showed unspecific regional slow rhythms. It is therefore advisable to always review these investigations if there is a specific suspicion of encephalitis.

Immunosuppression, including corticosteroids, intravenous immunoglobulins (IVIG), or plasma exchange, is standard treatment for anti-NMDAR encephalitis, possibly in combination. Neuroleptics should be used with caution. If the encephalitis is a paraneoplastic phenomenon, the tumor(s) must be removed. Patients who do not recover as expected can be given second-line immunotherapy with rituximab alone or in combination with cyclophosphamide [1]. Our patient’s condition gradually improved after initiation of immunosuppressive treatment and tumor removal, as demonstrated by repeated bedside cognitive assessments (Table). Possible side-effects from oral steroids could have been avoided with a faster taper.

Primary long-term sequelae of anti-NMDAR encephalitis is cognitive dysfunction, specifically in the domain of memory and executive functioning [9], but the role of young age in long-term outcomes remains unclear. In our patient, we could demonstrate improved cognition as measured by MMSE/MOCA-testing, paralleled by clinical improvement. Testing with MOCA and MMSE are tools widely used in investigation for dementia and, in recent studies reevaluating cognition after stroke, MOCA is suggested to be superior in detecting changes over time [12, 13]. This kind of simple testing could be helpful in monitoring symptoms in neuropsychiatric patients. Our patient also reported impaired vision at follow-up. As NMDARs are present in the retina [14], focal retinal dysfunction could explain this, or occipital cortical deficits could be responsible.

There should now be a low threshold in psychiatric patients for initiating investigations such as lumbar puncture, EEG, and neuroimaging, which makes demands on the organization of health services, as general anesthesia is often required and psychiatry services are not always located central to the hospital. Ultrasound or MRI of the abdomen should be considered in adolescent girls. If primary investigations with EEG and/or MRI produce negative results, these should be reviewed.

In conclusion, this case underlines the importance of collaboration between child and adolescent psychiatry and pediatrics. In our reported case, we arrived at the right diagnosis and treatment within 3 weeks of admission, which according to Wright is regarded as early (< 8 weeks) [15]. The initiation of a multidisciplinary discussion was a contributing factor; we suggest that adolescents with mania or psychosis who do not respond to mood-stabilizing treatment should always be subjected to further consultations and investigations. Psychiatrists and neurologists need to develop an integrated multidisciplinary approach to the assessment and management of brain disorders.

## Data Availability

Not applicable.

## Abbreviations

AFP: Alpha-fetoprotein
β-hCG: Beta human chorionic gonadotropin
CSF: Cerebrospinal fluid
CT: Computed tomography
ECT: Electroconvulsive therapy
EEG: Electroencephalography
ER: Emergency room
IVIG: Intravenous immunoglobulin
LP: Lumbar puncture
MDT: Multidisciplinary team
MMSE: Mini-mental state examination
MOCA: Montreal Cognitive Assessment
MRI: Magnetic resonance imaging
Anti-NMDAR: Anti-*N*-methyl-d-aspartate receptor
NMS: Neuroleptic malignant syndrome
